# American Dental Association

**Published:** 1891-10

**Authors:** 


### AMERICAN DENTAL ASSOCIATION.

The Thirty-first Annual Meeting of the American Dental Association was held at Saratoga Springs, N. Y., August 4 to 7, 1891.

*First Day.—Morning Session.*

Meeting called to order at 11.10 A.M., with the President, Dr. A. W. Harlan, in the chair.

Amendment to the constitution which was offered last year by Dr. Cushing was then read, as follows: "To amend by striking out the entire Section 2 of Article 4 of the Constitution."

Amendment adopted.

### REPORT OF THE PUBLICATION COMMITTEE.

The Publication Committee reported that in consequence of the S. S. White Dental Manufacturing Company having declined last year to publish the transactions, they were given to the *Dental Review*, of Chicago.

### REPORT OF THE TREASURER.

| | |
|---|---|
| Balance on hand, August 5, 1890 | $1368.74 |
| From dues to date | 1000.25 |
| Total | $2368.99 |
| Disbursements | 1175.00 |
| Balance | $1193.99 |

### REPORT OF THE EXECUTIVE COMMITTEE.

Dr. Crouse, on behalf of the Executive Committee, offered the programme for the convention.

### REPORTS OF COMMITTEES.

Report of Committee on Legislation and Appointment of Surgeons in the Army and Navy, read by Dr. Noble:

"The committee prepared a letter which was presented to the surgeon-general of the army and navy, as follows:

"'MARCH 27, 1891.

"'TO THE SURGEON-GENERAL OF THE UNITED STATES ARMY:

"'The undersigned committee was appointed by the American Dental Association, at its last meeting, to consider the feasibility of the appointment of dental surgeons in the army and navy. It is believed that the appointment of dental surgeons in the army and navy would be the means of relieving much suffering, and saving the organs of mastication so necessary to health and comfort. This want is especially felt in our Western military stations by both men and officers, far away from any dental surgeon, often sending members of their familes hundreds of miles to get the services of a dentist.

" 'We should be pleased to have your opinion and advice on the above subject.

" 'Very respectfully,

" 'A. H. Thompson, D.D.S., *Chairman*, Topeka, Kansas;
" 'J. Y. Crawford, M.D., D.D.S., Nashville, Tenn.;
" 'W. W. Walker, M.D.S., D.D.S., New York City, N.Y.;
" 'C. N. Peirce, D.D.S., Philadelphia, Pa.;
" 'H. B. Noble, D.D.S., Washington, D.C.,
" '*Committee.*'

"The letter was presented to the surgeon-general personally, with such statements of facts within our knowledge as were deemed important. The interview with the surgeon-general, Dr. Sutherland, was a very pleasant one. The following answer was received:

" 'War Department, Surgeon-General's Office,
" 'Washington, April 13, 1891.

" 'To the Gentlemen of the Committee of the American Dental Association:

" 'Gentlemen,—I have the honor to acknowledge the receipt of your communication of March 27, 1891, in which you suggest the appointment of dental surgeons in the army and navy, and request my opinion and advice on the subject. I am not in a position to speak as regards the navy, but so far as the army is concerned, it is a matter which has been considered by the War Department on several occasions. So long as our army is distributed in small bodies over a vast extent of territory, and is actually in the field for a considerable part of the year, it is deemed impracticable to extend to it the benefits of skilled dental surgery, however desirable this might be on behalf of officers and men. Where troops are massed in garrison, as in a few instances, the military reservation is, as a rule, in the vicinity of some large city, where the services of dental surgeons can be obtained.

" 'I am of the opinion also that the necessity for special dental service with the troops scattered over the West and South is less needful now than it was some years ago, for increased railroad facilities have brought the most remote posts within a few hours' journey of some great city.

" 'I have the honor to be, gentlemen,
" 'Respectfully yours,
" 'C. Sutherland,
" '*Surgeon-General, U.S.A.*'

"No response has been received from the surgeon-general of the navy, but the opinion was expressed that the need was growing less each year. We should therefore not have the encouragement or favor of the medical men of the army and navy. In asking or seeking such legislation at the hands of Congress, for it would require special law (act of Congress), we should be likely to encounter opposition, as the questions of rank, pay, residence, etc., would have to be considered.

"The men are only enlisted for a definite term of service, and are not accepted unless the mouth, in the judgment of the medical examiner, is in good condition and has enough good teeth to serve through the term of enlistment. The materials used by a dental surgeon would be a fruitful source of trouble in the way of expense. Officers would be very likely to want all sorts of impracticable work, and their families included on the free list, until the position of dental surgeon to 'Uncle Sam' would be far from pleasant or agreeable, sought or accepted only by the 'lazzaroni' of the profession, and would not be likely to elevate our profession in the estimation of the medical profession."

REPORT ON LEGISLATION, BY DR. H. B. NOBLE.

"WASHINGTON, D. C.

"The subject of dental legislation is a broad and deep one, and it is by no means clear just what is the best plan to secure unity of action in the various States, and also have sound principle for a basis of dental laws.

"There seems to be a diversity of opinion as to the powers that should be granted to examining boards of the various States, and whether a diploma from a reputable college shall exempt the holder from an examination. The trouble seems to be as to who shall determine what is a reputable college, and to draw a line between, or designate, the good and the poor, the reputable and the disreputable. It seems but fair and just that a graduate should have some standing, and, as a rule, we do not believe that examining boards are as well calculated to determine the competency of candidates as an instructor or regular teacher, and we have thought perhaps this difficulty could be overcome by a committee from the examining board in each State being present at the college examination in the State, and, if such examination were satisfactory to the committee, to endorse the diplomas, and when so endorsed they shall exempt the holder from any further examination.

"We think no reputable college would object to the presence and assistance of such a committee to endorse their work. Would not such a committee act as a stimulant to good college work, and also divide responsibility in the granting and rejecting of diplomas, and to disarm the charge of interested professors seeing how large a number they could graduate (that is sometimes heard of), and to make proficiency, rather than numbers, the standard of success in college work? A national committee selected from the National Association of Dental Examiners, might perhaps be better than a State committee, if such a committee could be appointed, for it is doubtful if competent national committees could be secured to serve without compensation.

"We believe in colleges and college work, and in the thoroughness of this must we look for the proper education, elevation, and growth of our profession. When their work has been properly done, let us endorse and encourage them by laws that will properly recognize their labor.

"By a national or State committee, working in harmony with our colleges, we shall best disarm the feeling of jealousy, or want of harmony, between college faculties and examining boards that has appeared at annual meetings of those two bodies and in various State societies.

"Let us, as an association, and as individuals, labor to harmonize the action of those bodies. In union there is strength! Can we not secure this union by a committee, either national or State, to assist in the examination of students? By such a union we should soon have harmony of action in our State laws. The National Association of Dental Faculties and the National Association of Dental Examiners have both performed a good and commendable work by securing a three-years' term of study and a more thorough and uniform system of examination of those who would enter our profession.

"When dental laws were first enacted, they did not contemplate the ignoring or supervision of a diploma from a reputable dental college; but the rapid increase of colleges and their competition for patronage soon brought doubt upon the thoroughness of the work of instruction in some of those schools, and to cast doubt upon a diploma as a test of fitness for practising our profession.

"It was then believed by many that the proper way to correct this was by giving power to examining boards to ignore all diplomas, which has been done in several States, thus reducing the reputable diploma from a good college to the level of the poorest, and casting a doubt upon all, which will soon be felt and acted upon outside of the United States.

"Is it not a better plan to seek to elevate and improve our colleges to the highest rank by recognizing their work when properly done? Is it not better to give their students standing and recognition, making a diploma mean what it says, rather than degrading and ignoring it?

"Have not our colleges furnished the men for society organization and work? Have not their societies been the parents of dental legislation and examining boards? We think they should seek, rather than discard, their teaching and advice.

"College training is one of the foundation-stones of professional life. Another reason for making our diplomas stand for something is that we may move our residence, and not be subjected to an examination from committees every time a State line is crossed. Some of our most skilful and scientific operators could not pass a strict technical examination, such as is required of students. Old practitioners, and those who have creditably passed an examination by those qualified to conduct it, should not, we think, be required to go before an examining board every time they may change their location. This may be avoided by recognizing a diploma from a reputable dental college. This, of course, brings us back to the character and reputation or standing of a college, and we think this can best be ascertained by a national or State committee.

"It does not seem feasible to seek a national law, as each State would justly claim the right to construct its own laws; but great care should be taken, in framing those laws, that they may be legal and just, and not retroactive, as in the case of the New Hampshire law that was overdrawn.

"Dental legislation has had a very rapid growth,—possibly too great for a sound and healthy stock. We think a little pruning of some of our State laws is desirable, rather than stimulation of growth, thereby rendering them more uniform and strong, and better adapted to the wants of the profession and to any assaults that may be made upon them, for it would be strange if assaults were not made upon them by those who may seek to avoid or overthrow them.

"Let us then seek to unite and strengthen our forces by united action of State and national societies, and of colleges and examining boards. In harmony and union there is strength and success!

"We wish to call attention to the fact of there being no dental law in the District of Columbia, and that the senators and representatives from the States have the making of the laws for the district, and that all dentists throughout the United States will ask their senators and members to give their attention to the matter which has been so long neglected.

"Year after year have bills been brought in only to be laid aside, and we ask that every member of this Association give this matter his attention and assistance."

The report was adopted by the Association and directed to be published in the transactions.

The committee on the New Hampshire State Board case then presented their report.

"The undersigned, appointed as a committee under the resolution adopted last year to investigate the nature and status of the litigation in regard to the dental law of New Hampshire, with authority 'in their discretion to draw on the treasurer of this Association for an amount not to exceed five hundred dollars,' would respectfully report as follows:

"That the statement made to this Association last year was correct as to the dental law of New Hampshire, passed in 1879, having been declared unconstitutional by the Supreme Court of that State. That the statement was incorrect as to the reported appeal of the case to the Supreme Court of the United States. The fact is that the dentists of New Hampshire wisely accepted the decision of their own highest court, and instead of wasting their means by an appeal to the Supreme Court of the United States, they applied themselves successfully to the enactment of a new law which does not contain the unconstitutional provisions.

"From the decisions of the New Hampshire Supreme Court it will be seen that the old law made an unjust, frivolous, and ridiculous distinction between men of the same class, and was retroactive in its most objectionable form. The distinction was that a dentist who had resided and practised his profession in the town or city of his residence January 1, 1879, for all the four years preceding its enactment, could retain his right to practise; but another man who, although he may have been in reputable practice in the city for fifty years, had moved either his place of business or his residence within the four previous years, lost all his rights.

"Your committee decided that it had nothing to do under the resolution but to report as above.

"Respectfully submitted,

"L. D. Shepard.

"Thomas Fillebrown.

"George H. Cushing."

The following is the decision in regard to the New Hampshire Dental Law.

At the adjourned law term of the Supreme Court, opinions were given in the cases of State *vs.* C. D. Hinman, and State *vs.* D. D. Pennoyer, the former a dentist, and the latter a physician, who were indicted in Rockingham County for practising without a license. The opinion in the first case was given by Judge Clark as follows:

"This is an indictment under Chapter cxxxii., 'General Laws,' for practising dentistry without a dental degree or license. The respondent demurred upon the ground that the statute is unconstitutional.

"The object of the statute upon which the indictment is founded is to secure the possession of the requisite skill and learning by practitioners of medicine, surgery, and dentistry. The possession of such special qualifications as to knowledge and skill is so essential to the protection of the health, lives, and comfort of the people of the State, that it cannot be doubted that it is within the power of the Legislature to secure it by the enactment of such reasonable conditions as are calculated to exclude from practice those unfitted therefor.

"Hewitt *vs.* Charier, 16 Pick. 353.

"State *vs.* State Medical Examining Board, 32 Minn. 324.

"Eastman *vs.* State, 7 West. Rep. 421 (Ind).

"Is the statute repugnant to the Federal or the State Constitution in any of its provisions? It is contended that it is in violation of Section 2 of Article 4 of the Constitution of the United States, and of Section 1 of the Fourteenth Amendment to the Constitution, because it discriminates against persons engaged in the same business or profession, and denies to them the equal protection of the laws, and that it is in violation of Article 2 of Part First of the State Constitution, which declares that all men have the natural and inherent right of acquiring and possessing property and seeking and obtaining happiness.

"Section 3 of Chapter cxxxii., 'General Laws,' upon which the indictment is based, provides that 'it shall not be lawful for any person who is not duly authorized to practise medicine or surgery to do so unless such person has secured a degree from some college or university authorized to confer the same, or shall have obtained a license from the New Hampshire Dental Society.'

"Section 6 provides that 'each person receiving a license upon examination shall pay, for the use of the society granting the same, the sum of five dollars; upon diploma, one dollar.'

"Section 3 declares that 'the provisions of the preceding sections shall not apply to persons who have resided and practised their profession in the town or city of their present residence during all the time since January 1, 1875, nor to physicians residing out of the State when called into the State for consultation with duly licensed physicians, or to attend upon patients in the regular course of business.'

"While the power of the Legislature to impose restrictions upon the exercise of certain trades and professions for the protection of the public is unquestioned, it must be exercised in conformity with the constitutional requirement that such restrictions must operate equally upon all persons pursuing the same business or profession under the same circumstances. The constitutionality of a statute cannot be sustained which selects particular individuals from a class or locality, and subjects them to peculiar rules, or imposes upon them special obligations or burdens from which others in the same locality are exempt.

"Cooley's Constitutional Limitations, 391.

"The imposition of special restrictions or burdens, or the granting of special privileges to persons engaged in the same business under the same circumstances is in contravention of the equal right which all can claim in the enforcement of the laws, and in the enjoyment of liberty, and the right of acquiring and possessing property.

"If the statute had declared that these provisions should not apply to persons practising their profession in the city of Concord, such an arbitrary discrimination would be clearly repugnant to the principles of constitutional equality.

"The exemption of all physicians, surgeons, and dentists residing and practising their profession in Concord from the burden of procuring and paying for a license, and the subjection of all other persons practising the same professions elsewhere in the State to the expense of purchasing a license, would be a palpable violation of constitutional rights.

"The exemption of the statute of persons who have resided and practised their profession in the town or city of their present residence during all the time since January 1, 1875, or during all the time from January 1, 1875, to January 1, 1879, from its operation, is no less in conflict with constitutional provisions.

"By an arbitrary test having no reference to skill, learning, or fitness for the practice of that profession, certain persons are exempted from the payment of a license fee, to which others of equal and perhaps superior acquirements and experience are subjected. It is a discrimination founded solely upon the accidental circumstance of residence, or of a change of residence, and falls within the prohibition of the constitution.

"The statute also discriminates against citizens of other States. It does not apply to persons residing and practising their profession in the same town or city in the State from January 1, 1875, to January 1, 1879, whence persons who have resided and practised their profession continually since January 1, 1875, in the same town or city, in another State, are required, upon removing, to procure another license to practise their profession.

"The objection to the statute is that it imposes the burden of a license fee upon certain persons and exempts others of the same class and profession, under similar circumstances and conditions.

"Loon Hing, *vs.* Crowley, 113 U. S. 703.

"Yick Wo *vs.* Hopkins, 118 U. S. 356.

"Demurrer sustained."

Dr. Fillebrown stated that he was authorized to express the thanks of the New Hampshire dentists for the kindly interest exhibited by the Association.

Dr. Taft offered a resolution, which was adopted, for the appointment of a permanent committee on necrology, to consist of three members, to act without further instruction from this Association as occasion may require, and report at the annual meeting of the society.

Committee on Credentials made a partial report.

### ADDRESS OF PRESIDENT HARLAN.

It is a time-honored custom, I believe, to have the President of this Association deliver an address, no matter how short or how long. Mine will be short.

The American Dental Association convenes to-day for its Thirty-first Annual Meeting. For the first time in its history we miss the familiar face and form of our lamented first-elected president, Dr. W. H. Atkinson, of New York, who died April 2, 1891. Dr. Atkinson was one of the most conspicuous figures in this body from the earliest period of its existence until our last annual meeting. It is not saying too much for him to say that he did as much, nay, more, for the building up of this Association than any single member whose name can be mentioned. It is a matter of personal sorrow to every one assembled here to find that his place is vacant, and that we shall see his face no more.

During the year other eminent and useful members of this profession have departed, the most notable being Dr. Maynard, of

Washington, a man who practised before dental colleges were established in this country, and who not alone gave his thought to the profession of his choice, but to other professions. He was a celebrated inventor, principally of fire-arms. He was a most progressive member of the society, and we feel that it has lost one of its most illustrious examples.

I must also mention Dr. White, the editor of the *Dental Cosmos.* Dr. White was a faithful member of this society, and attended the meetings in the capacity of a journalist, and to him we are indebted for the splendid line of transactions for years past. He was one of the few thoroughly well-qualified dental editors of this country. We speak the sentiment of the members of the society when we say his place cannot be filled either as a journalist or as head of the great house of which he had acted as president since the death of its founder.

The President feels that there are some topics which may be briefly touched upon relative to the usefulness and progress of this Association, and one of them is the method of doing work by committees. At one time it was thought best to leave the work to committees entirely. A glance at the transactions during that period will show that nearly all the work has devolved upon the chairman. The sectional system in an association as small as this seems to me unsuited to the best scientific work. It makes it incumbent for the chairman to assume the whole responsibility, and has a tendency to stifle individual workers. If there should be any personal ill-feeling existing between the members against the author of a paper, which I regret to say is sometimes the case, it often happens that papers are suppressed and never reach this body.

The President would respectfully recommend that a committee be appointed to inquire into the matter and see if an improvement to this effect cannot be made.

Another thing that the President would speak of, as affecting the interests of sections, would be this: that there should be a more general rotation in the filling of the office of president and secretary. It would increase the number of workers. I know that the chairman and secretary of some sections have been in office for many years, and it seems that this continual keeping in office has a tendency to detract from the usefulness of the members of such committees. It has happened in my own case. Although I have desired a rotation and the infusion of new blood, the members have refused, and I have filled that office with great reluctance. If it could be done with rotation, it would be a great benefit.

Another thing that was last year touched upon slightly was the method of doing the miscellaneous business of this Association. A committee was appointed on this subject. From the knowledge of the workings of the Odontological Association of Great Britain, the American Dental Association, the Illinois State Dental Society, and other societies in the United States, the plan of having an executive counsel perform all the strictly business work of the Association would seem a desirable one. Such a method leaves the society free to devote all its time to the reading of papers and the discussion of the same.

If the society saw fit to leave all its transactions to the Executive Committee of the Association, it seems to me that much more and better work would be accomplished.

In order to be a thoroughly American Dental Association, it should be possible for us to meet at any point in the United States, but under our present constitution it is impossible that this meeting should be convened at any point south of Mason and Dixon's line at the time fixed for our annual sessions. We recognize that there is a field for the creation of Western and Southern and also Eastern dental societies, but the one in which we are most interested should have its meetings at a time of the year when it would be most convenient to attend the sessions. As it is now, we must meet the first Tuesday of August in every year.

If you will consider this for a moment you will see that our membership would be greatly increased if we could go into a place where we have never been.

If the Association delegates its business to the Executive Committee, and gives them the power to effect this change, it is expected that the membership will be largely increased during the next ten years.

The question of increasing the interest in the annual meetings has been in my mind for a long time. Since a change has taken place in the political history of this country by the addition of new States, we should make effort to see that State dental societies are organized in every Territory and State now in existence. It would also be a matter of interest that local societies should be formed in towns and cities where it would be practicable,—in cities and towns of from twenty-five thousand to fifty thousand inhabitants. This is a matter to which I would call your especial attention, and urge immediate action.

At our last annual meeting a resolution was adopted appointing a general executive committee for the purpose of organizing the

World's Columbian Dental Meeting. By the generous distribution of funds as would be needed, we will accomplish a double object:

*First.* To secure a larger membership for local societies and State societies, and increase the interest in the same.

*Second.* By so doing we will assure for the projectors of the meeting full and cordial support.

During the past winter a meeting was held at Washington. Various committees were appointed, and they worked to make this a splendid success. It devolves upon you, gentlemen, to give this committee cordial support to secure not only the best efforts of our best men in the production of papers, but to make instructive clinics before the people of the world, and astound them with the progress of American dentistry and dental surgery.

I am sure that the most cordial co-operation will be given to this meeting from members of the profession residing in foreign countries. Every one agrees that it will be a convention of the most learned dentists of the world, and it seems that this would be an occasion never to be forgotten.

The Association has cause for congratulation to-day in the fact that at the beginning of the fall sessions a third-year course in our colleges becomes obligatory. It was agitated for some time past. Everything bids fair to make this new departure a firm fixture, and after a three-year course in one of the reputable colleges an American dentist will compare favorably with any dentist on this side of the water or on the other.

The Association is to be felicitated on its general progress.

A committee of five was appointed to take the President's address into consideration, and take such action on the recommendations therein contained as may be proper.

The Chair appointed Drs. Abbott, Barrett, Truman, Fillebrown, and Noble as the committee.

Adjourned.

